# Mitral Valve Prolapse and Sudden Cardiac Death in Athletes at High Risk

**DOI:** 10.2174/1573403X19666221220163431

**Published:** 2023-03-22

**Authors:** Olga Vriz, Irene Landi, Abdalla Eltayeb, Giuseppe Limongelli, Lucio Mos, Pietro Delise, Eduardo Bossone, Antonello D` Andrea

**Affiliations:** 1 Heart Centre, King Faisal Specialist Hospital & Research Centre, Riyadh, Saudi Arabia;; 2 Department of Translational Medicine, Università del Piemonte Orientale, Novara, Italy;; 3 Department of Translational Medical Sciences, Inherited and Rare Cardiovascular Disease Unit, University of Campania “Luigi Vanvitelli”, AORN dei Colli, Monaldi Hospital, Naples, Italy;; 4 Dipartimento di Cardiologia, Ospedale San Daniele del Friuli, Udine, Italy;; 5 Ospedale Pederzoli, Peschiera del Garda, Italy;; 6 Azienda Ospedaliera di Rilevanza Nazionale “A. Cardarelli” Hospital, Naples, Italy;; 7 Department of Cardiology, Umberto I Hospital, Luigi Vanvitelli University - Nocera Inferiore (ASL Salerno), Caserta, Italy

**Keywords:** Mitral valve, cardiac death, athletes, mitral valve prolapse, ventricular arrhythmias, premature ventricular contractions

## Abstract

Mitral valve prolapse (MVP) is the most frequent valvulopathy in the general population, with usually a favourable prognosis. Although it can be associated with some complications, ventricular arrhythmias (VA) and sudden cardiac death (SCD) are the most worrying. The estimated risk of SCD in MVP is between 0.2% to 1.9% per year, including MVP patients with and without severe mitral regurgitation (MR). The association between SCD and MVP is expressed by a phenotype called “malignant MVP” characterized by transthoracic echocardiography (TTE) findings such as bileaflet myxomatous prolapse and mitral annulus disjunction (MAD), ECG findings such as repolarization abnormalities, complex ventricular arrhythmias (c-VAs) and LV fibrosis of papillary muscles (PMs) and inferobasal wall visualized by late gadolinium enhancement cardiac magnetic resonance (LGE-CMR). Therefore, attention is raised for patients with “arrhythmic MVP” characterized from an ECG point of view by frequent premature ventricular contractions (PVCs) arising from one or both PMs as well as by T-wave inversion in the inferolateral leads. In athletes, SCD is the most frequent medical cause of death and in young subjects (< 35 years) usually is due to electrical mechanism affecting who has a silent cardiovascular disease and are not considered per se a cause of increased mortality. In MVP, SCD was reported to happen during sports activity or immediately after and valve prolapse was the only pathological aspect detected.

The aim of the present paper is to explore the association between SCD and MVP in athletes, focusing attention on ECG, TTE in particular, and CMR findings that could help to identify subjects at high risk for complex arrhythmias and eventually SCD. In addition, it is also examined if sports activity might predispose patients with MVP to develop major arrhythmias.

## 1 INTRODUCTION

Eligibility for competitive sports participation in subjects with valve disease is influenced by several factors such as symptoms, functional capacity, type and severity of valve disease, myocardial remodelling and function, pulmonary artery pressure and risk of arrhythmias. In asymptomatic individuals, mild valvular disease is considered safe and compatible with any type of sport. Although regular and continuous physical exercise is one of the strategies proposed in preventing cardiovascular disease, on the other hand, predisposes to sudden cardiac death (SCD) when occult heart disease is present [1]. The most frequent cardiovascular pathologies responsible for SCD in sports are hypertrophic cardiomyopathy in particular in North America [2], followed by arrhythmogenic right ventricular dysplasia (ARVD) in Italy and Spain [3]. Other causes are coronary artery anomalies, dilated cardiomyopathy, myocarditis, ion channel diseases, mitral valve prolapse (MVP), aortic dissection, short and long QT syndromes [4]. MVP is the most common valvular pathology with an estimated prevalence of 2-3% in the general population, usually considered a benign condition [5]. Nevertheless, MVP outcome varies, with a risk of MVP-related events at 10 years of 2% when no other major risk factors for cardiovascular mortality are present [6]. This valvular disease can be complicated by mitral regurgitation (MR) up to severe insufficiency with left ventricular (LV) remodelling or can be characterized by arrhythmogenic burden even in the presence of non-significant MR in a form called arrhythmic MVP (AMVP). Sudden cardiac death is an ultimate and rare complication of AMVP with an incidence of 0.14 events per 100 patients-years (95% CI 0.1 to 0.3) [7, 8]. Arrhythmic complications, symptoms such as syncope, cardiac arrest describe the “malignant MVP“ phenotype. AMVP is characterized by myxomatous, thickened mitral valve leaflets causing chordal elongation, leaflets prolapse into the left atrium and annulus abnormalities such as mitral annulus disjunction (MAD). The ECG is characterized by repolarization abnormalities, ventricular arrhythmias, premature ventricular beats (PVC) and eventually, ventricular fibrillation (VF). As confirmed by cardiac magnetic resonance (CMR), anatomy and electrophysiologic studies, the arrhythmic burden in AMVP correlates with fibrosis localized at the level of papillary muscles and basal segment of inferolateral wall of the left ventricle (LV), related to the prolapsing leaflets and annulus hypermobility.

The aetiology of SCD in competitive athletes is cardiovascular in 80-85% of cases, frequently arrhythmic (≤ 35 years old) [7, 9-11] and it is particularly dramatic because it happens in young subjects, otherwise healthy. At the moment, insufficient data are available on the association between MVP and SCD in athletes because this event is rare in the general population and even more in this particular cohort of subjects.

In the present paper, we reviewed the association between SCD and MVP in athletes, addressing those red flags that could help to identify a subgroup of subjects at high risk for complex arrhythmias and eventually SCD. Moreover, it was also investigated if sports activity might predispose patients with MVP to develop major arrhythmias.

## 2 DEFINITION OF MVP AND MITRAL APPARATUS ASSOCIATED ANOMALIES

### 2.1 Structural Characteristics of MVP

**Fig. (1):**
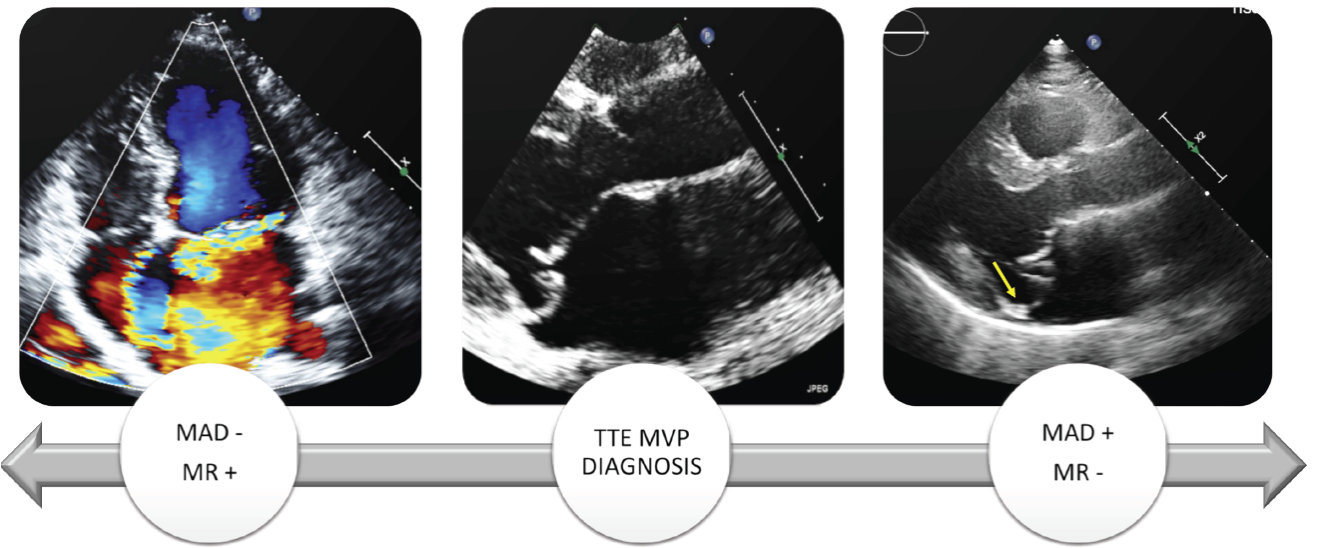
TTE diagnosis of MVP and its phenotypes. Moderate/severe MR is a complication associated with MVP with or without the presence of MAD, with different degree of LV remodelling, myocardial fibrosis and arrhythmias. MVP with trace-mild MR and MAD, bileaflet prolapse and curling, can have focal fibrosis (papillary muscles and basal segments of infero-lateral wall) that is a trigger of major arrhythmias. **Abbreviations:** MVP, mitral valve prolapse; MAD, mitral annular disjunction; MV, mitral valve; MR, mitral regurgitation; LV, left ventricle.

The suspicion of MVP is usually based on auscultation, characterized by mid-systolic click followed by a late systolic murmur if mitral regurgitation is present. The diagnosis is made by transthoracic echocardiography (TTE) in parasternal long-axis (PLAX) view [12]. In classic MVP, due to myxoid infiltration, leaflet thickness is at least 5 mm and leaflet prolapse in the left atrium is 2 mm or more. MAD can be associated with MVP, easily detected by TTE, with a prevalence that varies from 15% to 55% based on the different imaging modality used and population considered, strongly associated with myxomatous mitral valve disease [13-16]. MAD is visualized during ventricular systole as a distinct separation of the mitral valve annulus-left atrial wall and the basal region of the posterolateral LV myocardium, when the mitral annulus “slides” into the atrium, also defined “atrialization”, ranging from a few millimetres to more than 10 mm is the distance (Fig. **1**). A meta-analysis reported that MVP patients with MAD had a higher risk of VAs, with a risk ratio of 1.90 (*p* < 0.0001) [17]. Curling is another marker of MVP associated with arrhythmias. Curling is the unusual hypermobile outward and downward systolic motion of the posterior mitral annulus on the adjacent myocardium. Both of them are eventually associated with relative hypertrophy and fibrosis of the adjacent myocardium and should be included in the description of MVP disease [18, 19]. Importantly, MVP is a progressive disease.

### 2.2 Pathophysiology of Arrhythmic MVP (AMVP)

**Fig. (2):**
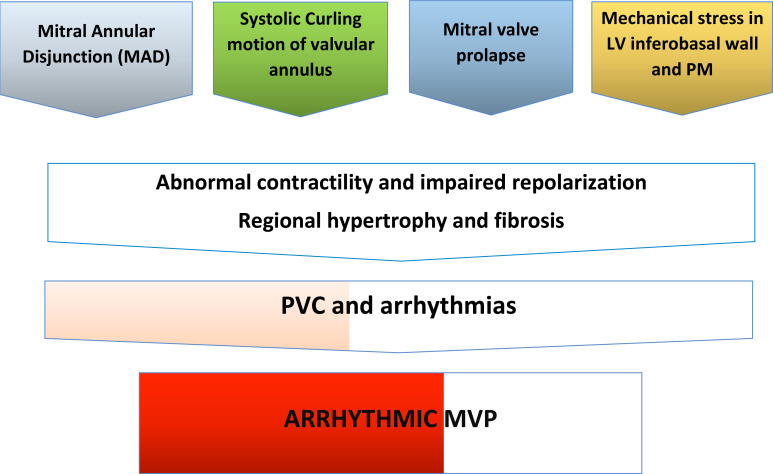
Pathophysiology of AMVP.

Combined with structural echocardiographic findings, AMVP could be additionally characterized by ECG findings such as T-wave inversion (TWi) in inferior leads, polymorphic/right bundle branch block (RBBB) morphology PVCs and c-VA. It is hypothesized that arrhythmogenesis in MVP has two components, a structural one, represented by cardiac fibrosis and a mechanical one based on abnormal tension on the papillary muscles (PMs) and wall stress on the adjacent LV basal segment of inferolateral myocardium (Fig. **2**). It has been shown in animal models that PMs traction by the prolapsing leaflets, changes per se the electrophysiologic function and prolongs ventricular functional refractory period locally [20], inducing sustained arrhythmias from PMs. MAD was also found to be associated with an increased frequency of premature ventricular beats (PVBs) and c-VA in comparison to those without MAD. Moreover, leaflets prolapse, MAD and curling are responsible for focal fibrosis documented post-mortem by histological analysis and *in vivo* by CMR imaging [21], leading to electrical instability. Electrophysiology studies mapped the site of origin of PVCs coming from PMs, LV outflow tract and mitral annulus, suggesting that the origin of arrhythmias is close to the prolapsing leaflets [20].

Also severe MR, as a complication of MVP, can contribute to the arrhythmogenic burden through negative LV remodelling (increased LV end systolic volume, increased LV mass and reduced LV ejection fraction) and increased myocardial fibrosis which are proportional to the degree of MR. Moreover, VAs are more common in MVP with fibrosis than in those without fibrosis [22]. Significant MR also predispose to increased pulmonary pressure and heart failure. In this case competitive sport activity is not recommended and clear guidelines are available [23]. In this paper this topic will be not discussed.

### 2.3 Autonomic Nervous System, Catecholamines and MVP

Patients with MVP could complain of palpitation, orthostatic rhythm disorders, exertional dyspnea, chest pain, syncope and neuropsychiatric disorders such as anxiety which are not related to mitral valve abnormalities and MR. It is hypothesized that these symptoms could be related to autonomic nervous system or neuroendocrine dysfunctions leading to sympathetic overactivity, reported significantly higher in MVP patients than in non-MVP ones [24]. MVP patients may have dysregulation of the sympathetic nervous system at the subcortical level, which explains the increase of sympathetic input into the cardiovascular system.

### 2.4 Genetics in MVP

MVP could be syndromic or non-syndromic. The syndromic variant is well recognized, and the clinical evaluation is focused on the syndrome and not on MVP itself. The most frequent syndromic disease associated with MVP is Marfan syndrome (MFS), a genetic disorder that affects the connective tissue caused by fibrillin-1 (FBN1) mutations in its most common form. In MFS, MVP prevalence increased over time, reaching 75% by the age of 60 years [25-28]. In MFS, mutations of the transforming growth factor-β (TGFβ) signalling pathway are also reported similarly to Loeys-Dietz syndrome, in which MVP is found in 25% of patients. Non-syndromic MVP is typically sporadic, but there are also familial/genetic patterns [29] where mutations in filamin A (FLNA) and Dachsous homolog-1 (DCHS-1) genes are involved [28]. Recently, exome slice sequencing analysis performed on unexplained SCD in the young (SUDY) with MVP confirmed by autopsy found a genetic variant of ryanodine receptor cardiac channel linked to arrhythmogenic and dilated cardiomyopathy [30]. Currently, genetic testing for non-syndromic MVP is not recommended due to the incomplete knowledge of the genetic background of inherited cardiovascular disease. In particular identification of gene variants of uncertain significance may lead to a misdiagnosis in the presence of inconclusive phenotypic features.

### 2.5 Risk of Sudden Death in a Sport Activity

Sudden cardiac arrest (SCA) in athletes is underestimated, and is identified only in 5-56% of cases. SCA usually occurrs during exercise (56-80% of cases) with an incidence higher in males than females with a ratio of 10:1 (RR 3:1 to 9:1) biased by the higher involvement of males in strenuous sports than females [2, 31]. More than 80% of all SCD is related to coronary artery disease in athletes >35 years of age, while little is known about the risk of cardiac death related to strenuous physical activity in young competitive athletes [23]. According to retrospective and registry studies, sudden death in competitive adolescents and young adults happened in subjects who were affected by silent cardiovascular diseases, congenital or acquired, and the main mechanism is electrical [2, 9, 32, 33]. Actually, sports activity is not considered per se a cause of the increased mortality but rather plays a role as a trigger [34].

### 2.6 MVP and SCD in Athletes

In the general population, the prevalence of MVP is higher in females [21, 35-37]. As aforementioned, in sport activity, males experience higher SCD than females [2, 31] including SCD associated with MVP, because males are more involved in sports. The estimated rate of SCD in the general population is 0.06-0.08%/year while MVP is 0.2%-1.9% per year including both MVP patients with LV dysfunction due to severe MR and MVP patients without significant MR [38]. There are some estimates that consider patients with MVP to have 3-fold risk to experience SCD than the general population [39] but probably is only slightly higher when MR is absent. In the athlete population, the prevalence of MVP is likely similar to the general population [33] and generally is not associated with hemodynamically significant MR. It is interesting to underline that several reviews on SCD in athletes do not mention MVP to be associated with SCD [31, 40-42] and when reported, MAD was not mentioned at all. Our knowledge on SCD and MVP in athletes is mainly based on registries and single centre studies (Table **1**).

Corrado *et al.* [43] analysed the Veneto Registry focusing on adolescents and young adults (12-35 years old) involved in competitive sports compared with a control group. Considering the period 1979-1999, 27 patients who died of SCD had MVP, and 6 (11%) were athletes (4 men, 66.7%; 2 women, 33.3%). MVP, being the 4^th^ cause, was more frequent pathology associated with SCD with a risk ratio of 3.2 in athletes than non-athletes, frequently occurring in men [9]. Maron and colleagues studied the prevalence of cardiovascular diseases causing sudden death in young athletes and compared these findings with a representative multicentre hospital based cohort of patients with HCM [44]. Among the 286 athletes who died for SCD, 9 (3%, 8 males and 1 female) had MVP, being the 10^th^ cause of cardiovascular mortality in this population. Caselli *et al.* [33] followed a cohort of 7449 competitive athletes for 8±2 years. MVP was described in 215 athletes (2.9%, 67% males) and MAD was mostly associated with VAs (16% *versus* 3%; *P*<0.001). Only athletes with MVP and VAs (8 subjects, 0.5%/y) had clinical events (mitral valve surgery, ischemic stroke, atrial fibrillation) while SCD did not occur during the follow-up period. Drezner *et al.* [45] analysed sudden cardiac arrest (SCA) in a large cohort of 1710 United States high schools that had onsite automated external defibrillators (AED) programs. In 36 subjects, 14 high school student athletes experienced SCA. No cases of SCA in MVP subjects were reported. Yanai *et al.* [46], from a retrospective forensic review, from 1980 to 1998, described 11.432 SCD, 36 (0.32%) during physical exercise, all of them were males except 1 female. The association between MVP and SCD was found in one case (3%). Another recent paper on athletes disqualified from sports because of ventricular arrhythmias that included MVP subjects did not report episodes of SCD during a 6-month follow-up [47].

At the moment, we do not know if there is an independent effect of MVP with or without MAD on SCD in sports. From the reports available, the likelihood of sports activity being a trigger for major VA and ultimately SCD in MVP seems to be low.

### 2.7 Risk Stratification for “Arrhythmic MVP” in Athletes: First Approach

In MVP, there are some features related to AMVP [33, 44] that must be investigated to rule out a potential arrhythmic risk. Also atrial fibrillation secondary to moderate/severe MR has to be carefully evaluated as a source of complex VAs as specified by guidelines [23].

#### 2.7.1 Anamnesis and Family History

Palpitation, dizziness, syncope of unknown aetiology, family history of sudden cardiac death in a person with MVP is a strong indication of AMVP. Han *et al.* [48] reported that in 161 cases of MVP with SCD or cardiac arrest, 79% had preceding symptoms, mostly palpitations. Moreover, SCD was associated with the situation of stress including physical activity (23% of cases) but in 46% of the cases, SCD happened during normal daily activities.

#### 2.7.2 Physical Examination

Usually MVP has no specific signs. At auscultation, a crisp mid-systolic click can be present and represents the prolapsing leaflet, associated with a late systolic murmur if MR is present. If syndromic MVP is suspected, the use of specific clinical scores can guide the diagnosis (Revised Ghent criteria [26] and Beighton 9-point scoring system [49], respectively for MFS and Ehlers-Danlos syndrome).

#### 2.7.3 ECG

Some ECG findings usually considered abnormal could be the result of physiological cardiac remodelling due to vigorous physical activity. For example, incomplete RBBB as well TWi from V1 to V4 in black athletes, ST segment elevation, and TWi in leads V1–V3 in athletes aged < 16 years should be reported as normal variants. Moreover, also TWi in leads V1–V2 in women does not warrant further evaluation in the absence of symptoms or family history of cardiomyopathy.

TWi ≥ 1 mm in depth in two or more contiguous leads in anterior, lateral, inferolateral, or inferior territory (except leads aVR, III and V1) is abnormal and the subjects should be further evaluated to rule out structural heart disease. Biphasic T waves or TWi in inferior leads (DII, DIII, aVF) are seen in MVP. In particular, TWi in inferior or antero-lateral leads is reported to be present in around 65% of subjects with bileaflet MVP and associated with VAs [premature ventricular contractions (PVCs), bigeminy, non-sustained ventricular tachycardia (NSVT), sustained ventricular tachycardia (VT) or ventricular fibrillation (VF)] [21, 36, 50, 51]. In the future ECG deep learning approach will help to increase the sensitivity to identify MVP at high risk to develop SCA [52].

A longer corrected QT interval is reported in MVP patients and associated with more leaflet prolapse and anterior mitral leaflet thickening and independently associated with VAs [50, 53]. QT dispersion (defined as the difference between the maximum and minimum QT intervals across all 12-leads of a single electrocardiogram) has been also correlated with VAs, MVP severity, and complex ventricular ectopy [54]. QTc can be more than 430 ms in MVP patients.

**Fig. (3):**
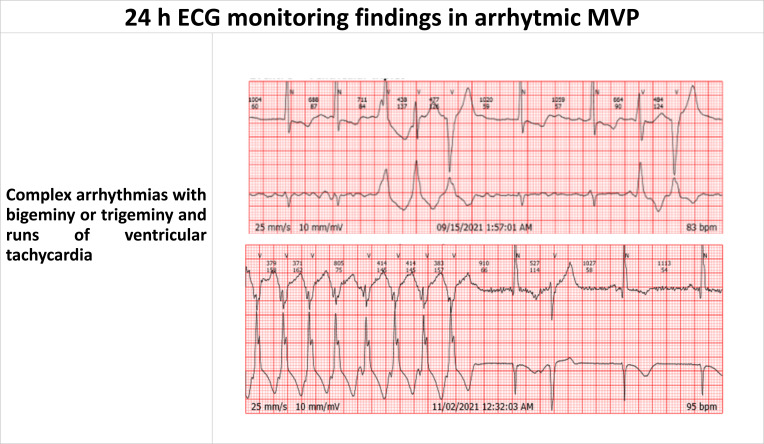
ECG findings in arrhythmic MVP.

PVC morphology is the most relevant feature to evaluate for the arrhythmic burden of MVP. Incomplete RBBB is common in young athletes and PVCs originating from the right ventricle outflow tract (left bundle branch block (LBBB) morphology, inferior origin) are considered benign unless they exceed 160 ms. In this case, can be an early sign of ARVD. PVC, with a burden more than 5% [50], and their complexity (PVCs, bigeminy, NSVT, VT or VF) are reported to be higher in MVP patients with SCD [36]. In MVP patients, the most frequent morphology is RBBB with a superior axis indicating papillary muscle origin or inferior axis originating from the inferolateral wall [21]. Some patients can also have LBBB. Actually, modern interpretation of PVCs in athletes mostly relies on morphology and their changes during exercise testing rather than their numerosity (Fig. **3** and Table **2**) [55].

#### 2.7.4 24-hour ECG Holter

**Fig. (4):**
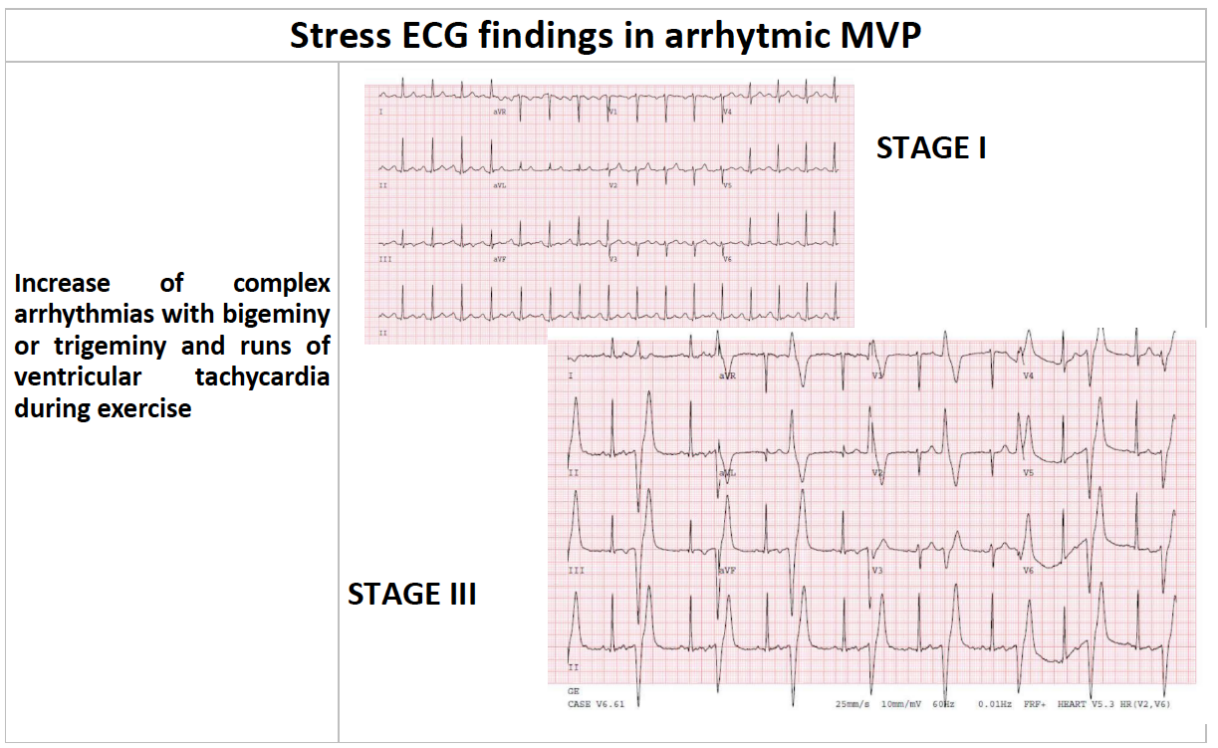
24 h ECG monitoring findings in arrhythmic MVP.

As aforementioned, malignant arrhythmic events do not rely on number of PVCs [56, 57] and Lown grading system, that do not correlate with the amount and localization of fibrosis detected by CMR, but rather PVCs morphology [58]. Usually, 24-hour ECG Holter is done using 2-3 leads and PVCs morphology cannot be described. For this purpose, it is suggested to use 12 leads recording. However, 24-hour ECG Holter is important for recording c-VA such as bigeminy, VT and NSVT. Chest strap devices could be also used for monitoring athlete`s maximal heart rate and HR irregularity while they are engaged in intense sports activity (Fig. **4** and Table **2**) [59].

#### 2.7.5 Physical Stress Test

**Fig. (5):**
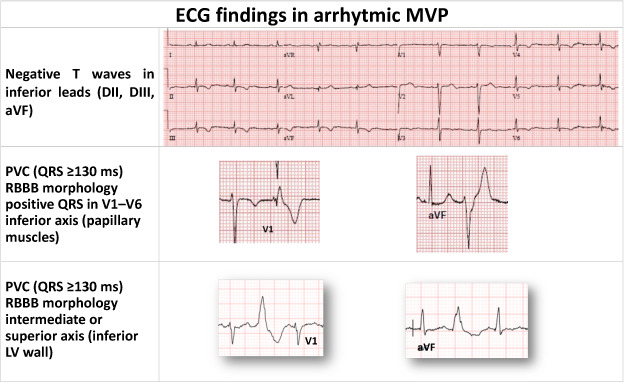
Stress ECG findings in arrhythmic MVP.

Exercise is pro-arrhythmogenic, causing mainly PVCs and non-sustained VT and is able to detect silent arrhythmogenic disorders. If physical examination and rest ECG identify cardiac abnormalities at risk of SCD in 0,28% of screened athletes, physical stress test increments of a further 0.21%, incrementing the sensitivity of athletic preparticipation evaluation by 75% [60]. The physiology of exercise is characterized by vagal tone suppression, even before the exercise starts, and sympathetic activation that leads to increased heart rate and ventricular contraction. Exercise testing should be maximal and carried out until muscular exhaustion, not just limited to 85% of the predicted maximal heart rate. Several studies have demonstrated that PVCs morphology and their changes during exercise can predict the presence of focal LV fibrosis by CMR [58, 60, 61], and may be the sole manifestation of cardiac disease at risk of SCD during sports activity. This is the case of pathological myocardial substrate in athletes in general, but also in athletes with MVP. On the other hand, Basso *et al.* [21] reported that some of their MVP-SCD cases had negative physical stress test (Fig. **5** and Table **2**).

#### 2.7.6 TTE

The diagnosis of MVP is made by echocardiography. In addition to classic mitral valve morphologic description (mitral regurgitation severity, left ventricular remodelling, systolic function, *etc*.), specific TTE red flags should be investigated to evaluate the arrhythmic risk in MVP. These are long redundant and prolapsing leaflets, MAD, systolic curling of the posterior MV annulus, high lateral S’ on TDI (> 16 cm/s, Pickelhaube Sign) and focal longitudinal strain [19, 39, 62]. In particular, MAD per se is considered a marker of AMVP but there is no consensus on cut-off severity. Mantegazza *et al.* [63] reported that MAD by CMR < 4 mm is frequently underdiagnosed by TTE but still has high arrhythmic risk. Carmo *et al.* [15] reported that MAD > 8.5 mm by TTE was a reasonable criterion to predict the risk of VAs with a sensitivity of 67% and a specificity of 83%. MAD diagnosis requires evaluation through the entire cardiac cycle and often has to be evaluated frame by frame. MAD is part of the mitral apparatus involvement and its presence does not necessarily mean focal fibrosis, as its absence does not exclude fibrosis when significant valve prolapse and redundant leaflets are present (Table **3**).

#### 2.7.7 Cardiac Magnetic Resonance (CMR)

*CMR* imaging is an excellent tool to study the mitral valvular apparatus, leaflets thickness, prolapse severity and particularly for myocardium characterization. In addition, it is more specific than transoesophageal echocardiography (TEE) and TTE in detecting the presence of MAD [62, 63]. Late gadolinium enhancement (LGE) images, which are usually obtained after 10 minutes of gadolinium contrast injection, identify areas of fibrosis at papillary muscles and the basal segment of the inferolateral wall just behind the PML. It has been shown that these focal areas of fibrosis are the foci of origin of arrhythmias [18, 21, 62]. Patients with MR and MVP have more focal fibrosis than those with only MR [64]. CMR has also shown a disproportionate LV remodelling and fibrosis in patients with trace-mild MR who have a higher probability of complex arrhythmias and worst outcome suggesting a specific MVP-related myocardial disease [22]. Recently, Dello Russo *et al.* [47] found that the best predictors of sports ineligibility at 6-month follow-up among athletes with VAs, including MVP patients, were syncope, abnormal ECG, LGE at CMR and electroanatomic mapping (Table **3**).

**Fig. (6):**
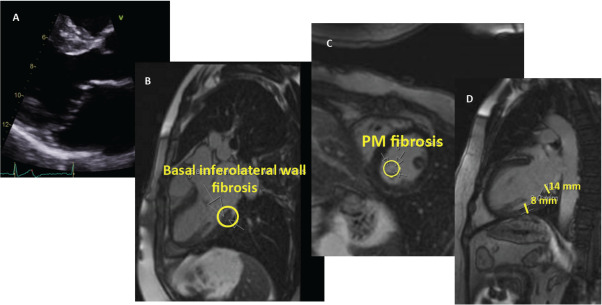
Patient with MVP, redundant leaflets and no MAD (panel **A**). CMR LGE findings of basal inferolateral wall and PM fibrosis (panel **B** and **C**) with basal/mid segment ratio > 1.5 (panel **D**).

Although it is not routinely performed, there are emerging data that myocardial extracellular volume (ECV) quantification by T1 mapping is an accurate tool to detect ECV expansion, a quantitative marker of diffuse myocardial fibrosis and myocardial damage. Pavon *et al.* [65] found that ECV was higher in MVP patients with MAD compared with those with MVP-No MAD and MR and those with only MVP. Moreover, ECV was similar among patients with MVP and MAD, regardless of the presence or absence of LGE. The authors also found that MVP patients with out-of-hospital cardiac arrest, had higher LGE and ECV (Fig. **6**).

#### 2.7.8 Cardiac Computed Tomography (CCT)

CCT is another useful modality for the evaluation of mitral valve anatomy and MAD but does not allow tissue characterization or MR severity quantification, so it is rarely utilized. Toh *et al.* [66] defined different phenotypes of MVP, such as the cases of fibroelastic deficiency and forme fruste of MVP demonstrated less extensive disjunction than Barlow disease. It is worth mentioning that the same authors described the presence of MAD in 96% of patients without heart disease [67].

### 2.8 MVP Management in Athletes

Most of the time, MVP does not imply any sports restriction. In the case of a) history of syncope or family history of SCD at young age; b) long QT; c) TWi in inferior leads on ECG; d) moderate to severe MR; e) PVCs with RBBB, supraventricular or ventricular tachycardia with RBBB at rest or during the stress test, athletes are disqualified from sports and should undergo CMR for focal fibrosis according to European Society of Cardiology (ESC) guideline on Sport Cardiology [68] and Italian Screening for fitness to sport [69].

The recent consensus document from the European Heart Rhythm Association (EHRA) on MVP and MAD [70] in the general population proposed an approach for risk stratification and management, keeping in mind the incomplete knowledge of physiopathology and clinical outcome, cost-effectiveness and resources, and avoid unnecessary and even harmful interventions.

There are not validated scores to help stratify the arrhythmic risk in MVP. The management of this valvulopathy in athletes has to be personalized, knowing that the prevalence is the same in the general population, is a progressive disease [33, 71-73] and more important, sports can be a trigger of major arrhythmias when an occult pathological substrate is present such as focal myocardial fibrosis [47, 74-81]. A possible approach is outlined in Fig. (**7**). In case of MVP, the first step is detailed family and personal history, in particular syncope that per se is an indication for further evaluation (other significant symptoms are palpitation and shortness of breath). Physical examination should be focused on signs of syndromic-MVP and heart murmurs and clicks. Resting ECG, 24-hour Holter and physical stress test will be done for the evaluation of arrhythmogenic burden and evidence of specific markers of AMVP. TTE will not only define the diagnosis and complications of MVP (mitral regurgitation, *etc*.) but will also focus on predictors of arrhythmias such as MAD, annular curling, the extent of leaflet prolapse, chamber size and function, LV strain, and tissue Doppler velocities. Although there is no evidence yet, we think that in the grey zone of low arrhythmic risk on ECG, athletes with TTE red flags have to be evaluated with particular attention. If cardiac CMR is abnormal or the initial history and testing is highly suspicious for an arrhythmic variant, the athlete will be considered not eligible for participating in sports and further specific evaluation will be planned [47].

## CONCLUSION

MVP is a common valvulopathy usually characterized by a benign course. Asymptomatic athletes with mitral valve prolapse with no red flags for arrhythmogenic variant can participate in all competitive and leisure sports with annual evaluations including physical examination, ECG, 24-hour Holter, exercise stress testing, TTE to obtain eligibility as per guidelines. In the case of MVP and severe MR, subjects may compete in low- to moderate-intensity sports after detailed evaluation according to guidelines. If severe MR is associated with characteristics of arrhythmogenic MVP, multimodality imaging techniques should provide a broad spectrum of information for further management and will follow valvular disease guideline indications. The decision making is more complex for those athletes with no significant MR and high-risk features for AMVP. An individualized approach is of paramount importance to identify that subgroup of athletes with MVP and with the cluster of features (symptoms, bileaflet redundancy, MAD, rest and dynamic ECG changes, echocardiographic red flags) that require further evaluation and appropriate management. Moreover, it seems that sport does not lead to SCD in MVP athletes per se, as in the case of ARVD but can trigger arrhythmias. On the other hand, it is crucial not to overemphasize the arrhythmic burden in MVP cases, the majority of that, do not have the criteria of malignancy, considering the potential effect of MVP diagnosis on young healthy subjects that want to be engaged in sports activity as a tool of social interaction. Much remains unknown regarding MVP, “arrhythmic MVP“ and the most fearful “malignant MVP“ type in general and in the population of athletes in particular.

## Figures and Tables

**Fig. (7):**
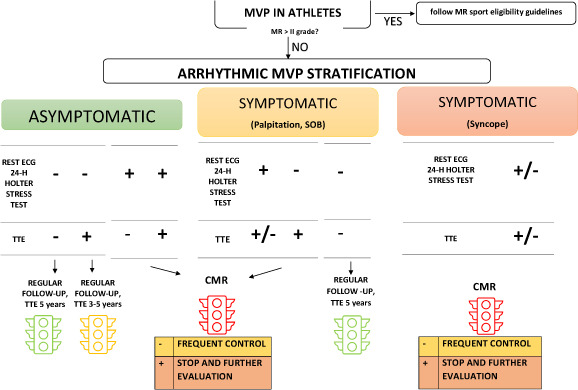
MVP management in athletes. After clinical evaluation, if TTE shows severe MR (MR > grade II), eligibility and management should be considered following guidelines. In the other cases, a complete electrocardiographic and echocardiographic arrhythmic MVP stratification should be done. In asymptomatic athletes, regular annual follow-up with clinical evaluation, ECG, 24-hour Holter and stress test should be performed when the first electrical risk assessment is negative (Table **2**). TTE should be repeated at three or five years depending on the presence of echocardiographic red flags (Table **3**). CMR has to be planned when electrical and/or TTE highlighted features indicating AMVP (Tables **2** and **3**). The presence of fibrosis at papillary muscles and at the basal segment of the inferolateral wall by CMR requires further investigation such as electrophysiology study (EPS) and electroanatomical mapping (EAM), following a prespecified protocol at highly specialized centres. If CMR is negative, eligibility status should be redefined at 6-month follow-up. In symptomatic athletes (palpitations, shortness of breath) CMR is recommended when electrical and/or TTE characteristics are suggestive of high arrhythmic burden. In case of history of syncope, CMR should be performed even in absence of electrical or TTE red flags. **Abbreviations:** MVP, mitral valve prolapse; MR, mitral regurgitation; TTE, transthoracic echocardiography, CMR, cardiac magnetic resonance; AMVP, arrhythmic mitral valve prolapse.

**Table 1:** MVP and sudden cardiac death in athletic populations.

**Author and Year of Publication**	**Study Period**	**Population**	**Total of Cases**	**Methods**	**Exertional/Non Exertional**	**Incidence** **(Deaths per 100 000 Person-Years)**	**Event**	**Cause of Death**	**Autopsy**	**Cases of SCD Attributed** **to MVP**	**Sex of SCD in MVP**	**MAD**	**MR**
Corrado *et al*, 1990 [77]	*1979-1989*	Young competitive athletes in Veneto region aged 11-35 years	22 M 19 F 3	Retrospective	Exertional 16 Non exertional 2	?	SCD	ARVD 6 Atherosclerotic CAD 4 Conduction system pathology 3 AOCA 2 MVP 2 Other 5	Yes	2 (9%)	M 1 F 1	?	?
Van Camp *et al.*, 1995 [78]	*1983-1993*	U.S high school and college sports aged 17-24 years	100 M 92 F 8	Retrospective	All exertional	0.7	SCD	HCM 56 AOCA 16 Myocarditis 7 Aortic Stenosis 6 DCM 5 Atherosclerotic CAD 3 Non specific cardiomiopathy 2 Subaortic stenosis 2 Coronary artery aneurism 1 MVP 1 Aortic Rupture 2 RVC 1 WPW 1	Yes	1 (1%)	M 1 F 0	?	?
Maron *et al.*, 1996 [10]	*1985-1995*	Young competitive athletes aged 12-40 years	141 M 120 F 21	Retrospective	Exertional 164 1 h after exercise 78 During mild physical activity or at rest 11 During sleep 2	?	SCD 141 (7 CC)	HCM 48 Probable HCM 14 AOCA 17 Other coronary anomalies 8 Ruptured aortic aneurysm 6 Tunnelled LADCA 6 Aortic valve stenosis 5 Myocarditis 4 Idiopathic dilated cardiomyopathy 4 ARVD 4 Idiopathic myocardial scarring 4 MVP 3 Atherosclerotic CAD 3 Other congenital heart syndrome 2 Long QT syndrome 1 Sarcoidosis 1 Sickle cell trait 1 ‘Normal’ heart 3	Yes	3 (2%)	?	?	?
Corrado *et al.*, 1998 [79]	*1979-1996*	Young Competitive athletes in Veneto region aged 11-35 years	49 M 44 F 5	Prospective	Exertional 35 Non exertional 5	1.6	SCD	ARVD 11 Atherosclerotic CAD 9 AOCA 6 Disease of conduction system 4 MVP 5 HCM 1 Myocarditis 3 Myocardial bridge 2 PTE 1 Dissecting aortic aneurysm 1 Dilated cardiomyopathy 1 Other 5	Yes	5 (10%)	?	?	?
Corrado *et al.*, 2003 [9]	*1979-1999*	Competitive athletes aged 12 to 35 years in the Veneto region of Italy	55 M 50 F 5	Prospective	Exertional 40 1 h after exercise 9 Non exertional 6	1.5 in M 0.5 in F	SCD	ARVD 12 Atherosclerotic CAD 10 AOCA 7 MVP 6 Myocarditis 5 Conduction system pathology 4 Myocardial bridge 2 HCM 1 DCM 1 LQTS 1 Mechanical causes 2 Other 7	Yes	6 (10,9%)	M 4 F 2	?	?
Maron *et al.*, 2003 [44]	*1985-2000*	Young Competitive athletes aged 9 to 40	286	Retrospective	Exertional 204 1 h after exercise 129 During formal athletic contest 75 During mild recreational physical activities 82 Non exertional 25 During sleep 11	?	SCD	HCM 102 Coronary artery anomalies 37 Possible HCM 29 Myocarditis 20 Ruptured aortic aneurysm 12 ARVD 11 Tunnelled coronary artery 11 Aortic valve stenosis 10 Atherosclerotic CAD 10 DCM 9 MVP 9 Coronary artery hypoplasia 8 Other congenital coronary anomalies 8 Cardiac sarcoidosis 3 LQTS 3 Congenital heart disease 3 Myocardial infarction 1	Yes	9 (3,15%)	M 8 F 1	?	?
Corrado *et al.*, 2006 [43]	*1979-2004*	Competitive athletes aged 12 to 35 years in the Veneto region of Italy	55	Prospective	During exercise 50 1 h after exercise 5	1.9	SCD	Cardiomyopathies 14 CAD 11 Cardiac Conduction Disease 4 Myocarditis 7 Coronary anomalies 7 MVP 6 Other 6	Yes	6 (10,9%)	M 5 F 1	?	?
Maron *et al.*, 2009 [2]	*1980-2006*	U.S. competitive organized team or individual athletes aged 8-39 years	1049	Retrospective	Exertional 844 Non exertional 205	?	SCD/D	HCM 251 Coronary Anomalies 119 Possible HCM 57 Myocarditis 41 ARVD 30 Ion channel Disease 25 MVP 24 LADCA brigde 23 CAD 23 Aortic rupture 19 AS 17 DCM 14 WPW 11 Other 36	Yes	24 (2,3%)	M 20 F 4	?	?
Marijon *et al.*, 2011 [80]	*2005-2010*	Competitive athletes aged 10 to 35 years old General population aged 10-75 performing physical activity – (non competitive,)	820 50 in competitive athletes (6%)	Prospective	All Exertional or 1 h after	Non Competitive 0,8 Non competitive 0,2	SCA/D	Acute Coronary Syndrome 152 HCM 7 Possible HCM 6 Congenital Cardiac Abnormality 5 Dilatated cardiomiopathy 5 Myocarditis 5 ARVD 3 Arrhytmias 4 MVP 2	Yes	2 all in the competitive population (4%)	M 2 (100%) F 0 (0%)	?	?
Maron *et al.*, 2014 [81]	*2002-2011*	Athletes of National Collegiate Athletic Association aged 17-26 years	47 SCD	Retrospective	Exertional and non exertional	1.2/100000	SCD	HCM 21 Coronary Artery Anomaly 8 Atherosclerotic Coronary Disease 5 Aortic Dissection 3 ARVD 3 Myocarditis 2 Dilated Cardiomyopathy 2 MVP 1 Acute Myocardial Infarction 1	Yes	1 (0,02%)	Male 1 (100%)	?	?

**Abbreviations:** CAD, Coronary Artery Disease; HCM, Hypertrophic Cardiomyopathy; ARVD, Arrhythmogenic Right Ventricular Dysplasia; AOCA, Abnormal Origin of Coronary Artery; DCM, Dilated Cardiomyopathy; CC, Commotio Cordis; RVC, Right ventricular cardiomyopathy; WPW, Wolf Parkinson White; LADCA, Left Anterior Descending Coronary Artery; PTE, Pulmonary Thromboembolism; LQTS, Long QT Syndrome; AS, aortic stenosis.

**Table 2:** Electrical risk stratification for AMVP in athletes.

**ECG and 24-h ECG Holter**
Corrected QT interval > 430 ms [54]
Short T-wave inversion in inferior leads (DII, DIII, aVF) [21]
PVC morphology: RBBB (QRS>130 ms) with superior axis (inferior wall) or indetermined/inferior axis (papillary muscles) [21]
PVC coupling (< 350 ms) [51]
Monomorphic PVC [36]
Polymorphic PVC: constant beat to beat change in morphology configurations of outflow tract alternating with papillary muscle or fascicular origin [36]
NSVT > 100 beats/min and lasting < 30s [36]
SVT lasting > 30s or requiring termination [36]
**Physical Stress Test**
ECG changes during stress test. Couplets/non-sustained ventricular tachycardia during the initial phase (first and second minute), at peak exercise or during post exercise [55]
Burden of complexity of VAs [50]: - Low risk: Frequent PVCs with no high risk morphology and not complex arrhythmias (No complete RBBB). - Intermediate risk: Polimorphic PVCs, bigeminy, trigeminy couplets, NSVT (heart rate less than 180 bpm). - High risk: SVT, polymorphic NSVT, NSVT with heart rate more than 180 bpm. Proven history of VF.

**Abbreviations:** PVC, Premature Ventricular Contraction; NSVT, Non Sustained Ventricular Tachycardia; SVT, Sustained Ventricular Tachycardia; VA, Ventricular Arrhythmias; RBBB, Right Bundle Branch Block; VF, Ventricular Fibrillation.

**Table 3:** Multimodality imaging red flags in AMVP: echocardiographic and cardiac magnetic resonance characteristics. The parameters in red are those most significant for the identification of high likelihood of AMVP and should be reported.

**TTE Findings**
Bileaflet MVP, entity of prolapse
Myxomatous degeneration (leaflets length and thickness)
Mitral annulus disjunction (MAD)
Systolic curling of the posterior annulus of MV
High lateral S’ on TDI (> 16 cm/s) (Pickelhaube Sign)
Basal/mid segment of inferolateral wall ratio >1.5
Paradoxical movement of the mitral annulus (systole bigger than diastole)
Dilated annulus
**CMR Findings**
Left ventricle size and function
Mitral regurgitation severity stratification
MAD, curling, annulus diameter and leaflets characteristics
Assessment myocardial fibrosis and localization (papillary muscles and basal segment of the inferior wall)

## LIST OF ABBREVIATIONS

PMs: Papillary Muscles
MR: Mitral Regurgitation
VA: Ventricular Arrhythmias
MVP: Mitral Valve Prolapse
SCD: Sudden Cardiac Death
TTE: Transthoracic Echocardiography
MAD: Mitral Annulus Disjunction
PVCs: Premature Ventricular Contractions
